# Increasing Participation in a TelePrEP Program for Sexual and Gender Minority Adolescents and Young Adults in Louisiana: Protocol for an SMS Text Messaging–Based Chatbot

**DOI:** 10.2196/42983

**Published:** 2023-05-31

**Authors:** William Richard Traylor Braddock, Manuel A Ocasio, W Scott Comulada, Jan Mandani, M Isabel Fernandez

**Affiliations:** 1 Department of Pediatrics School of Medicine Tulane University New Orleans, LA United States; 2 Department of Psychiatry and Biobehavioral Sciences David Geffen School of Medicine University of California, Los Angeles Los Angeles, CA United States; 3 Office of Public Health Louisiana Department of Health New Orleans, LA United States; 4 College of Osteopathic Medicine Nova Southeastern University Fort Lauderdale, FL United States

**Keywords:** chatbot, conversational agent, develop, iterative, messaging, text message, HIV, PrEP, pre-exposure prophylaxis, user testing, rule-based, prevention, eHealth, telehealth, mobile phone, sexual minority youth, gender minority youth, young adult, youth, adolescent, sexual minority, gender minority, gender diverse, gender diversity, SMS, artificial intelligence, patient education, health information, web-based information, user experience

## Abstract

**Background:**

Sexual and gender minority (SGM) adolescents and young adults (AYAs) are at increased risk of HIV infection, particularly in the Southern United States. Despite the availability of effective biomedical prevention strategies, such as pre-exposure prophylaxis (PrEP), access and uptake remain low among SGM AYAs. In response, the Louisiana Department of Health initiated the LA TelePrEP Program, which leverages the power of telemedicine to connect Louisiana residents to PrEP. A virtual TelePrEP Navigator guides users through the enrollment process, answers questions, schedules appointments, and facilitates lab testing and medication delivery. To increase the participation of SGM AYAs in the program, the TelePrEP program partnered with researchers to develop a chatbot that would facilitate access to the program and support navigator functions. Chatbots are capable of carrying out many functions that reduce employee workload, and despite their successful use in health care and public health, they are relatively new to HIV prevention.

**Objective:**

In this paper, we describe the iterative and community-engaged process that we used to develop an SMS text messaging–based chatbot tailored to SGM AYAs that would support navigator functions and disseminate PrEP-related information.

**Methods:**

Our process was comprised of 2 phases: conceptualization and development. In the conceptualization phase, aspects of navigator responsibilities, program logistics, and user interactions to prioritize in chatbot programming (eg, scheduling appointments and answering questions) were identified. We also selected a commercially available chatbot platform that could execute these functions and could be programmed with minimal coding experience. In the development phase, we engaged Department of Health staff and SGM AYAs within our professional and personal networks. Five different rounds of testing were conducted with various groups to evaluate each iteration of the chatbot. After each iteration of the testing process, the research team met to discuss feedback, guide the programmer on incorporating modifications, and re-evaluate the chatbot’s functionality.

**Results:**

Through our highly collaborative and community-engaged process, a rule-based chatbot with artificial intelligence components was successfully created. We gained important knowledge that could advance future chatbot development efforts for HIV prevention. Key to the PrEPBot’s success was resolving issues that hampered the user experience, like asking unnecessary questions, responding too quickly, and misunderstanding user input.

**Conclusions:**

HIV prevention researchers can feasibly and efficiently program a rule-based chatbot with the assistance of commercially available tools. Our iterative process of engaging researchers, program personnel, and different subgroups of SGM AYAs to obtain input was key to successful chatbot development. If the results of this pilot trial show that the chatbot is feasible and acceptable to SGM AYAs, future HIV researchers and practitioners could consider incorporating chatbots as part of their programs.

**International Registered Report Identifier (IRRID):**

PRR1-10.2196/42983

## Introduction

### Background

Sexual and gender minority (SGM) adolescents and young adults (AYAs) continue to be at increased risk of HIV infection [1]. The stigma of being a sexual or gender minority and the concomitant isolation, rejection, racism, marginalization, and discrimination experienced by these youth contribute to their elevated risk of HIV acquisition [2,3]. Focal points of new HIV cases among SGM are New Orleans and other southern municipalities where 50% of the new HIV cases among SGM are being diagnosed [4]. Biomedical prevention strategies, particularly pre-exposure prophylaxis (PrEP) that reduces the risk of acquiring HIV by 92%-99% [5-9], are highly efficacious. Yet, PrEP uptake is low among SGM AYAs [10]. To realize the full benefits of PrEP, we must increase access and continued engagement in PrEP services by individuals most at risk of acquiring HIV, especially SGM AYAs residing in the South.

In response to this need, the Louisiana Department of Health initiated the Louisiana (LA) TelePrEP Program in 2019. LA TelePrEP provides a novel and convenient way for Louisiana residents 18 years of age or older to access PrEP through telemedicine. A TelePrEP Navigator guides users through the enrollment process, answers questions, schedules virtual medical appointments, and facilitates lab testing and medication delivery. The Navigator also facilitates access to ancillary services including insurance enrollment. The entire enrollment process can be done remotely by completing the self-enrollment form accessible on the LA TelePrEP website [11] or doing so with support from the Navigator. Unfortunately, participation of SGM AYAs in the Program is limited. From 2018 to 2020, only 52 SGM AYAs had contacted the Program, and only 12 were below the age of 21 years. Increasing the reach of the LA TelePrEP services to SGM AYAs is vital to curbing the further spread of HIV in this community. In 2020, the LA TelePrEP Program partnered with researchers from the Adolescent Medicine Trials Network in efforts to increase participation of SGM AYAs in the LA TelePrEP Program. This collaboration aims to extend the Program’s current outreach efforts and support the Navigator’s functions by developing a chatbot (PrEPBot) and PrEP-focused social media messaging campaign targeting SGM AYAs.

### Chatbots for Public Health

A chatbot is an automated system that mimics informal human-to-human conversation [12]. Chatbots can vary greatly in technical sophistication and capabilities. Artificial intelligence (AI)–based chatbots use natural language understanding and machine learning to provide conversational, contextualized responses (ie, human-like) based on input from users. Simpler chatbots can be programmed using rule-based conversations that are scripted but have limited capacity for recognizing free text input. Many companies provide programmable communication tools to develop application programming interface chatbots that can be integrated with various messaging platforms. Chatbots have been widely adopted in education, e-commerce, consumer banking, entertainment, travel, transportation, and logistics, and their use has led to documented benefits for both employers and customers [13]. Chatbots can be accessed around the clock and reduce employee workload by scheduling appointments, setting reminders, answering questions, locating services, and disseminating information. These functions transfer to health care and public health settings [14]. Chatbots have also been used as interventionists. For instance, Woebot is a “talk therapy” chatbot embedded within Facebook Messenger that has been shown to reduce symptoms of depression and anxiety [15,16].

Despite the wide use of chatbots in other public health areas, the migration of chatbots to HIV prevention efforts has been slow [17]. In 2020, The Joint United Nations Program on HIV/AIDS launched Eli [18,19], a chatbot designed to engage youth in text-based conversations focused on issues critical to healthy youth development such as family, relationships, and sexual health. Eli addresses HIV prevention by providing reasons why testing is important, where and how to get tested, and the importance of starting treatment if HIV test results are positive [18]. The initial response suggests that this type of service targets an important need. In the first week alone, over 12,000 users engaged with the service, and Eli answered over 150,000 questions [18,19]. Eli’s success supports exploring text-based chatbot options for other prevention strategies, such as PrEP, among SGM AYAs. Chatbots may help overcome the stigma and discrimination often faced by SGM AYAs by providing a medium to communicate sensitive information and connect to prevention services privately [20]. With extensive community engagement, an SGM AYA–targeted PrEPBot could provide concise, high-quality information, respond to requests rapidly, have 24-hour access, and connect interested users with either a TelePrEP Program Navigator or the enrollment website to learn more.

### Goal of This Protocol

In this paper, we describe the iterative process that we used to develop an SMS text messaging–based chatbot tailored to SGM AYAs that would support Navigator functions and disseminate PrEP-related information. Lessons learned and suggestions that researchers could consider in future application of SMS text messaging–based chatbot technology for HIV prevention efforts were also included. The process we describe in this paper was conducted as part of a parent study designed to increase participation of SGM AYAs in the Louisiana TelePrEP Program. The parent study consisted of a web-based survey and structured focus groups whose findings were used to develop the chatbot and the targeted social media campaign. The institutional review board (IRB) approval for the study included permission to beta-test the chatbot. The testers were not considered to be research participants since their function was to provide recommendations and feedback regarding the functionality of the chatbot. In a separate paper, we will describe the development of the social media campaign and report the findings.

## Methods

### Phase I—Conceptualization

Prior to chatbot development, the research team met with the TelePrEP Navigator weekly to discuss job responsibilities, program logistics, and interactions with users to identify tasks that would ideally be programmed into the chatbot. The discussions were guided by the following questions: (1) What are your primary responsibilities as a TelePrEP Navigator? (2) What tasks take up most of your time? (3) If you could gain another “human” team member, what would you want them to be able to do? (4) What are the main reasons people contact the TelePrEP Program? and (5) What are the most common questions that people ask you about PrEP and the TelePrEP Program?

Through these conversations, we learned that some of the most time-consuming elements of the navigator’s role were discussing PrEP eligibility, facilitating the enrollment process, scheduling appointments, identifying and overcoming PrEP access barriers (eg, insurance), and answering general questions. At our meetings, concerns about the chatbot seeming overly robotic and using inaccessible language that would deter our target population from engaging with the chatbot were also shared.

It was decided that an AI-based chatbot would best meet our functional needs while being “personable.” After further investigation, it was realized this type of chatbot required considerable technical expertise, financial resources, and time that were beyond the scope of the project. Instead, we opted to use a rule-based chatbot that was still capable of carrying out all of the functions that would improve Navigator efficiency. A rule-based chatbot was more appropriate for the scope of the project and could be developed using commercially available application programming interface chatbot platforms, such as Twilio [21]. We hired an undergraduate computer science student and proceeded with development using Twilio.

### Phase II—Iterative Community–Engaged Development Process

#### Overall Process

**Figure 1:**
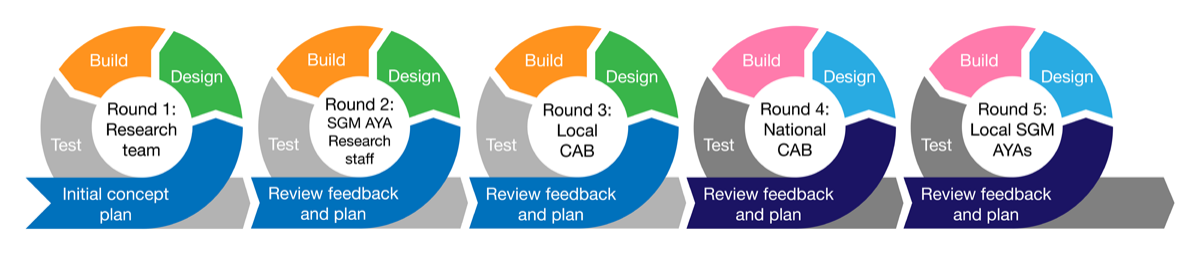
A diagram of the iterative community–engaged development process. AYA: adolescents and young adults; CAB: community advisory board; SGM: sexual and gender minority.

To balance the need to program functions that would support the Navigator while ensuring that the chatbot was user-friendly and accessible to our target population, principles of user-centered design were used to guide our development process [22]. We intentionally engaged Department of Health staff and SGM AYAs within our professional and personal networks. Our iterative process involved multiple stages of testing starting with those directly involved with the project, and each sequential stage approaching real-world conditions to better evaluate the functionality of the chatbot (Figure 1).

In the initial testing phases, we developed unique profiles of hypothetical users from information gathered during the conceptualization phase (see Textbox 1). Each tester was assigned a different profile to use when interacting with the chatbot. Through these conversations, the testers identified areas in need of improvement, technical glitches, typos, and issues with language and terminology.

**The unique profiles of hypothetical users assigned to testers during the initial testing phase.**

**Scenario A**

- You want to get on pre-exposure prophylaxis (PrEP)
- You’re 18 years old
- You have private insurance
- You’re on your parents’ insurance
- You don't have any questions

**Scenario B**

- You want to get on PrEP
- You’re 16 years old
- You have public insurance
- You’re on your own insurance plan
- You don’t have any questions

**Scenario C**

- You want post-exposure prophylaxis (PEP)
- You’re 20 years old
- You were exposed 24 hours ago
- You already have a doctor you see

**Scenario D**

- You want PEP
- You’re 29 years old
- You were exposed 4 days ago
- You don't have a doctor already

**Scenario E**

- You want to know if you're eligible for PrEP
- You’re 26 years old
- You're not living with HIV
- You’ve had anal sex recently
- You're unsure of your partner’s status
- You don’t always use condoms
- If you’re eligible, you don’t want to get on PrEP today
- If you’re ineligible, you don't have any more questions

**Scenario F**

- You have questions about PrEP
- You want to know how PrEP works
- You also want to know if you have to take it everyday

We used 3 groups of testers in the initial testing phase. First, the research team, other HIV research staff, and a local community advisory board (CAB) evaluated the functionality of the prototype chatbot. The research team was comprised of 2 HIV researchers, 1 senior biostatistician, 1 research coordinator, an undergraduate computer science student, and the TelePrEP Navigator. Our second group of testers were 3 SGM AYA research staff who worked on other HIV-focused projects. Third, the prototype was tested by 8 members of a Louisiana-based youth CAB who were identified as SGM AYA and had a working knowledge of the project.

In the later testing phase, SGM AYAs who had no familiarity with the project were engaged. We convened 2 groups to provide feedback on the chatbot’s functionality. The first was comprised of 8 members of the ATN National CAB, and the second consisted of 5 SGM AYAs recruited from our networks. At the start of each testing session, we provided a brief description of the TelePrEP Program and then instructed the participants to freely engage with the chatbot. No additional instructions were provided. After each iteration of the testing process, the research team met to discuss feedback, guide the programmer on incorporating modifications, and test the chatbot to ensure feedback had been properly integrated.

#### Initial Development

Our first charge was to create flow diagrams to provide a visual representation of anticipated conversations between the chatbot and potential users. We relied heavily on the navigator’s experiences interacting with TelePrEP contacts as the foundation for designing the structure and content of these conversations. We focused our initial efforts on PrEP eligibility, facilitating the enrollment process, scheduling appointments, and identifying and overcoming PrEP access barriers (eg, insurance).

**Figure 2:**
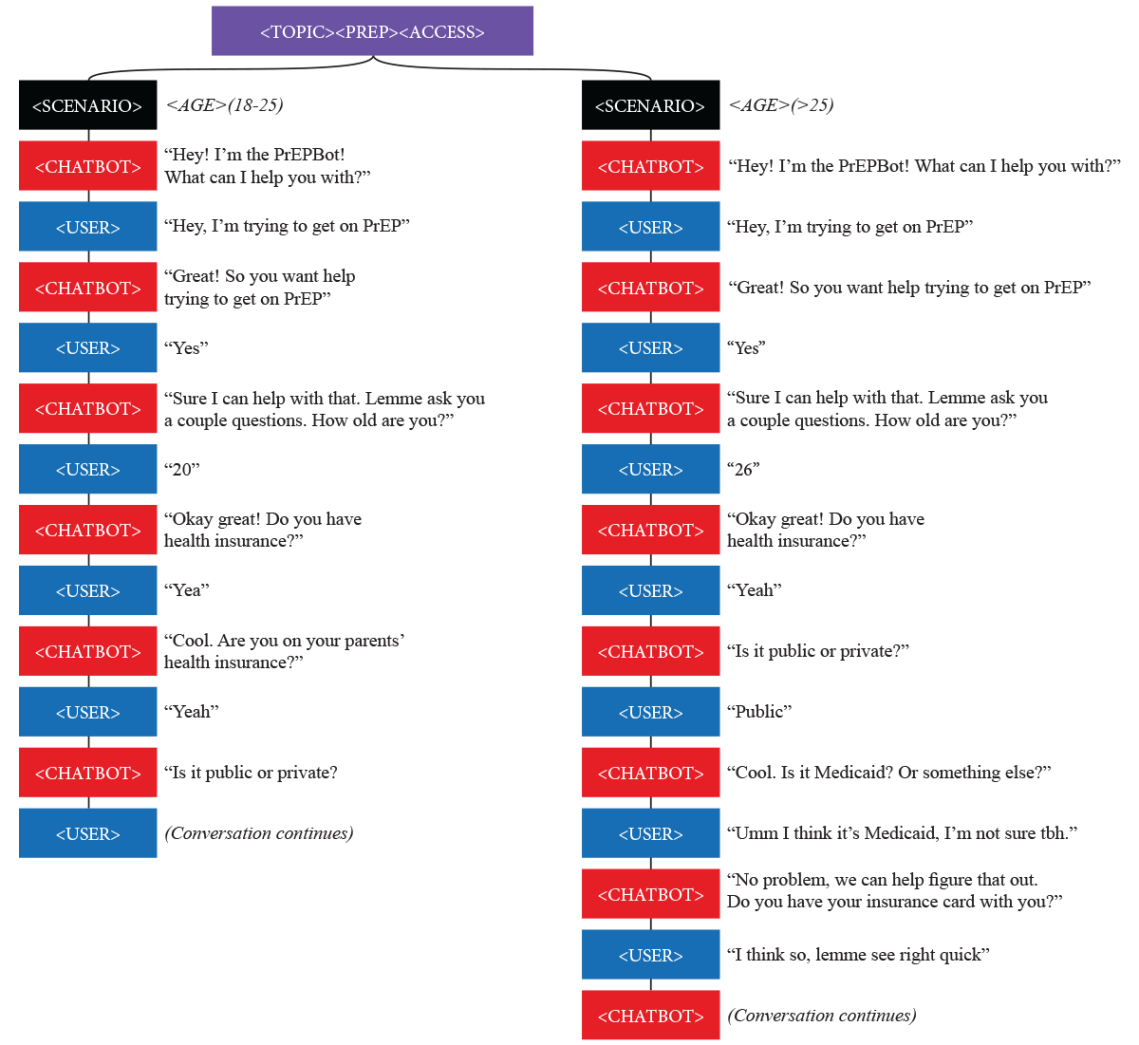
Initial chatbot conversation flow diagram. PrEP: pre-exposure prophylaxis.

Flow diagrams were created using presentation software (Figure 2) that the programmer would then translate to the Twilio platform using JavaScript coding. Conversation flows were designed based on relevant user characteristics and intent. For example, age and insurance status were important considerations for both program eligibility and addressing privacy implications for those on parents’ insurance. In addition to the flow diagram, we created a document to list all conceivable variations of user input in each step of the conversations (eg, yes, yeah, sure, and yup) to minimize the chatbot misunderstanding or misinterpreting communications. We incorporated scheduling appointments with a navigator by inserting a link that directed users to a Calendly calendar [23].

We initiated our iterative testing process as described earlier. After each testing session, the team discussed feedback and proposed solutions that we would route back to the programmer to make the modifications.

#### Programming Refinements and Ongoing Development

Our initial approach proved efficient for programming straightforward conversations that had limited variation in response options. PrEP eligibility, program enrollment, and scheduling conversation flows were easy to design since users could only respond yes or no. It was quickly realized that dichotomous responses could not be used to answer general program and PrEP-related questions. As we began to program the most commonly asked questions, we noted the nearly infinite ways the questions could be posed and the strong potential for typos, abbreviations, and colloquialisms that, if not entered verbatim, the chatbot would not understand. Furthermore, as the volume of conversation flows became more expansive and iterative changes more frequent, we realized that programming was inefficient and time-consuming. We needed to find an alternative approach for chatbot development.

Twilio tech support advised us to take advantage of additional tools in their platform that did not require a skilled programmer, specifically Studio and Autopilot. Studio is a visual workflow builder that uses drag-and-drop tools as well as custom code for rapid chatbot development. Our research coordinator, who had no experience with programming or chatbots, quickly learned how to navigate Studio and was able to easily make modifications to the chatbot that previously could only be handled by the programmer. We relied on the Autopilot tool to facilitate programming of commonly asked questions. Autopilot is an AI platform that can identify and respond to free text input and could be integrated into our existing rule-based design structure.

Once we incorporated the most common questions and formulated initial responses, we continued our iterative development process by engaging with the National ATN CAB and SGM AYAs in our personal networks. Through this process, the list of questions was expanded, and our responses were revised. This was especially important for colloquial use of certain terms. We could not feasibly integrate all possible questions and variations in wording of preprogrammed questions. Autopilot was able to understand slight variations in question wording and allowed us to program new variations as testers introduced additional ways to pose questions. In the event the chatbot could not understand a question, users would be sent to a frequently asked questions (FAQ) page on the TelePrEP website. Our final round of SGM AYA testers had minimal recommendations for changes and gave overall positive feedback on their experience. Through our highly collaborative and community-engaged process, we successfully created a rule-based chatbot with AI components to improve TelePrEP Navigator efficiency and increase access to the TelePrEP Program among young SGM AYA.

### Ethical Considerations

The parent study which included the beta testing described in this paper was approved by the Tulane IRB (2021-207). The role of the testers was to evaluate the chatbot to improve its functionality. They were not considered to be research participants.

## Results

### Recommendations for Chatbot Development

During our iterative development process, we gained important knowledge that could advance future chatbot development efforts for HIV prevention among SGM AYAs. In this section, we identify issues that hampered the user experience (UX) and describe our strategies for resolving them.

- Sending too many messages for a single response: Testers complained that many chatbot messages were too long, and they preferred concise responses in as few messages as possible. When feasible, it was programmed one response per message ensuring that responses were 160 characters or less, the limit for a single SMS text message.
- Asking unnecessary questions: The conversation flow was streamlined removing redundancies and unnecessary questions or branches. For example, we initially programmed questions that facilitated the enrollment process but were unnecessary to achieve the goal of linking the user to the TelePrEP Program. For instance, we had initially programmed the chatbot to ask the user for their insurance information. However, the user would have to provide this information to the navigator when enrolling, making this question redundant and unnecessary (Figure 2).
- Responding too quickly: Though chatbots are lauded for their ability to immediately respond, testers expressed that receiving responses so quickly was jarring or too robotic. A 5-second delay was programmed to more closely mimic the natural flow of texting conversations.
- Misunderstanding user input: As is common with all chatbots, testers encountered situations in which the chatbot could not interpret user input. In the initial stages of development, the chatbot would continuously ask for clarification when it did not understand a user’s input. Users became annoyed when the chatbot continued asking for clarification after a number of failed attempts. We programmed the chatbot to direct the user to the navigator after 2 consecutive failed attempts to understand the user’s input.
- Directing users to external links: Directing users to external links discouraged them from continuing to interact with the chatbot. Whenever feasible, the chatbot was programmed to provide information directly to the user. For instance, programming responses to the myriad of possible user questions was overwhelming, so we initially directed the user to our FAQ page. To reduce the number of times users were directed to the FAQ page, responses were programmed that could address multiple questions, and users were only directed when absolutely necessary (see Textbox 2).

**Using the Autopilot tool within Twilio, similar questions were assigned single responses that could more appropriately answer users’ queries.**

**Question group 1**

- Can I stop using condoms?
- How effective is pre-exposure prophylaxis (PrEP) at preventing infection?
- How effective is PrEP at preventing HIV?
- How effective is PrEP?
- Is PrEP effective?
- How well do doctors trust PrEP?
- Does PrEP really prevent HIV?
- Why should I take PrEP?
- Why take PrEP?
- Does it work well?
- How well does PrEP prevent HIV?
- How well does PrEP work?
- How good is PrEP?
- Does PrEP work?

**Combined answers 1**

- PrEP is highly effective when taken as indicated. The once-daily pill reduces the risk of getting HIV from sex by more than 90%. Among people who inject drugs, it reduces the risk by more than 70%. (Your risk of getting HIV from sex can be even lower if you combine PrEP with condoms and other prevention methods).

**Question group 2**

- Will hormone replacement therapy interact with PrEP?
- Will my medicine interact with PrEP?
- Will PrEP interact with other pills I take?
- Does Descovy interact with other medicine?
- Does Truvada interact with other medicine?
- Does PrEP interact with other medicine?
- What about poppers?
- Can I use mushrooms while I'm taking PrEP?
- Can I mix cocaine and PrEP?
- Can I use poppers while I’m on PrEP?
- Do poppers interact with PrEP?
- Does molly interact with PrEP?
- Can I take molly while I'm on PrEP?
- Does marijuana interact with PrEP?
- Does cocaine interact with PrEP?
- Can I smoke weed while I'm on PrEP?
- Does PrEP interact with marijuana?
- Does PrEP interact with weed?
- Can I use drugs when taking PrEP?
- Can I do drugs and take PrEP?

**Combined answers 2**

- Recreational drugs are not known to interact with either Truvada for PrEP or Descovy for PrEP. While PrEP generally has few interactions with prescription medicine, please consult your PrEP provider for information about how PrEP interacts with other medications.

## Discussion

### Principal Findings

This project demonstrates that a small team of behavioral researchers and staff can create a functional SMS text messaging–based chatbot to support a state-sponsored TelePrEP Program. We learned that it was important to dedicate sufficient time to the conceptualization process to ensure that the chatbot’s functions would address the needs of the Navigator and efficiently link the users to the TelePrEP Program. Our iterative process of engaging researchers, Program personnel, and SGM AYAs to obtain input was key to our success.

We will pilot the chatbot in tandem with the launch of the social media messaging campaign. To evaluate the acceptability of the chatbot, a survey was created based on an existing chatbot evaluation with some modifications [24]. These questions assess users’ demographics, intent of engaging with the chatbot, as well as their experiences using the chatbot. Chatbot users will receive a link to the 5-minute Qualtrics survey at the end of a chatbot conversation or, for incomplete conversations, a message with the link will be sent 15 minutes after the last interaction. Participants will be compensated US $5 for completing the survey. We will monitor usage and identify glitches or other unaccounted for issues that can be addressed when refining the chatbot. We will publish the results of this pilot trial and lessons learned in a subsequent manuscript.

### Implications

The implementation of chatbots and AI tools within messaging platforms has paralleled the surge of mobile phone use among AYAs [25,26]. However, chatbots are relatively new to the public health arena. A 2022 scoping review assessing the research base for the use of chatbots in public health identified the need for more research describing the development process and evaluating the efficacy and effectiveness of these chatbots [25]. The breadth of information shared in both this paper and an upcoming paper evaluating the feasibility and acceptability of the PrEPBot will begin to address this gap in the literature.

Community engagement was an essential component of our chatbot development process. Community members helped us to refine the chatbot language to be accessible and familiar to the end user as recommended by Barreto et al [27] in their study of chatbots for promoting child health. They also helped us to identify the type of questions users would ask and the information to be provided. With their assistance, we were also able to identify strategies to create a streamlined experience. For example, the number of “failed responses” was limited, so if the system failed to provide a direct answer after 3 attempts, it would automatically triage the user to the navigator [28]. Another important consideration was to ensure that the information provided by the chatbot was reliable and valid [28]. To develop the FAQ section, we drew upon publicly available information from trusted sources, such as the US Centers for Disease Control and Prevention [29] and the San Francisco AIDS Foundation [30]. Participation from the community helped us to incorporate information in the FAQs that resonated with and was relevant to SGM AYAs [27,31].

In developing chatbots for public health, programming and end user fees must also be considered [27]. We opted for SMS text messaging because of its low cost and wide accessibility. A readily available, easily deployable platform was used to program and host our cloud-based, chatbot conversation flow. We also noted that aside from labor, development costs were negligible. There was no charge to use the platform aside from the charges associated with sending and receiving SMS text messages during testing sessions, which cost US $60. We hope the PrEPBot’s inherent value as a low-cost SMS text messaging–based chatbot rather than as an independently designed app with a custom UX design will increase accessibility and ease of maintenance.

### Limitations

There were factors that limited our chatbot development process. Limited budget and technical expertise did not allow us to create a chatbot with advanced AI and natural language processing capabilities that could enhance the UX and better meet user needs. Though our community-engaged and iterative process was a major strength, we could have incorporated additional stages of testing to further refine the chatbot prior to launch.

### Conclusions

Chatbot development tools are accessible, user-friendly, and relatively inexpensive. An undergraduate computer science student and a research coordinator with no prior experience could successfully oversee programming and testing. Our experience proves that HIV prevention researchers can feasibly and efficiently program a rule-based chatbot with the assistance of commercially available tools.

## Abbreviations

AI: artificial intelligence
AYA: adolescents and young adults
CAB: community advisory board
FAQ: frequently asked question
IRB: institutional review board
PrEP: pre-exposure prophylaxis
SGM: sexual and gender minority
UX: user experience

## Data Availability

No research data were generated in the development of the chatbot.
